# Linking Power Doppler Ultrasound to the Presence of Th17 Cells in the Rheumatoid Arthritis Joint

**DOI:** 10.1371/journal.pone.0012516

**Published:** 2010-09-01

**Authors:** Nicola J. Gullick, Hayley G. Evans, Leigh D. Church, David M. Jayaraj, Andrew Filer, Bruce W. Kirkham, Leonie S. Taams

**Affiliations:** 1 Centre for Molecular and Cellular Biology of Inflammation, King's College London, London, United Kingdom; 2 National Institute for Health Research Comprehensive Biomedical Research Centre, Guy's and St. Thomas' Hospital and King's College London, London, United Kingdom; 3 Rheumatology Department, Guy's and St Thomas' NHS Foundation Trust, London, United Kingdom; 4 Rheumatology Research Group, Division of Immunity and Infection, University of Birmingham, Birmingham, United Kingdom; Singapore Immunology Network, Singapore

## Abstract

**Background:**

Power Doppler ultrasound (PDUS) is increasingly used to assess synovitis in Rheumatoid Arthritis (RA). Prior studies have shown correlations between PDUS scores and vessel counts, but relationships with T cell immunopathology have not been described.

**Methodology/Principal Findings:**

PBMC were isolated from healthy controls (HC) or RA patients and stimulated *ex vivo* with PMA and ionomycin for 3 hours in the presence of Golgistop. Paired synovial fluid (SF) or synovial tissue (ST) were analysed where available. Intracellular expression of IL-17, IFNγ, and TNFα by CD4+ T cells was determined by flow cytometry. Synovial blood flow was evaluated by PDUS signal at the knees, wrists and metacarpophalangeal joints of RA patients. Serum, SF and fibroblast culture supernatant levels of vascular endothelial growth factor-A (VEGF-A) were measured by ELISA. The frequency of IL17+IFNγ-CD4+ T cells (Th17 cells) was significantly elevated in peripheral blood (PB) from RA patients vs. HC (median (IQR) 0.5 (0.28–1.59)% vs. 0.32 (0.21–0.54)%, p = 0.005). Th17 cells were further enriched (mean 6.6-fold increase) in RA SF relative to RA PB. Patients with active disease had a higher percentage of IL-17+ T cells in ST than patients in remission, suggesting a possible role for Th17 cells in active synovitis in RA. Indeed, the percentage of Th17 cells, but not Th1, in SF positively correlated with CRP (r = 0.51, p = 0.04) and local PDUS-defined synovitis (r = 0.61, p = 0.002). Furthermore, patients with high levels of IL-17+CD4+ T cells in SF had increased levels of the angiogenic factor VEGF-A in SF. Finally, IL-17, but not IFNγ, increased VEGF-A production by RA synovial fibroblasts *in vitro*.

**Conclusions/Significance:**

Our data demonstrate a link between the presence of pro-inflammatory Th17 cells in SF and local PDUS scores, and offer a novel immunological explanation for the observation that rapid joint damage progression occurs in patients with persistent positive PDUS signal.

## Introduction

The pathogenesis of Rheumatoid Arthritis (RA) is complex and involves multiple cells and inflammatory mediators, including monocytes/macrophages, fibroblasts, B cells and T cells [1]. A large body of literature, in both animal models of experimental arthritis and human disease, demonstrates the critical contribution of pro-inflammatory CD4+ T helper cell subsets to pathogenesis, with particular roles for their signature cytokines IFNγ (produced by Th1 cells), IL-17A (produced by Th17 cells) and TNFα (produced by both Th1 and Th17 cells, as well as monocytes/macrophages) (reviewed in [2]).

Multiple animal models have demonstrated key roles of IL-17A (henceforth called IL-17) and Th17 cells in the immunopathology and joint damage of arthritis (reviewed in [3]). Further evidence for the role of IL-17 in RA is provided by its biological properties *in vitro* and *in vivo*, as it induces monocyte- and fibroblast-derived pro-inflammatory cytokines (TNFα, IL-1β, IL-8), mediators of bone and cartilage damage such as matrix metalloproteinases and RANK-ligand (RANKL) [4], [5], [6], neutrophil and monocyte recruitment [7], [8], and osteoclastogenesis [9]. Bone and synovial explants from RA joints exhibit increased production of functional IL-17 compared to OA joints [10], and IL-17+ CD4+ T cells can be found in RA synovial tissue [11], [12]. In early RA the cytokine profile of synovial fluid (SF) is dominated by the presence of IL-17 as well as Th2 cytokines [13]. Significantly, recent clinical trials with IL-17 antagonists in established RA show up to 42% ACR50 responses with short duration therapy [14], providing the first direct evidence in humans of a pathological role of IL-17 in RA, and indicating that blockade of IL-17 in humans may be a valid therapeutic approach.

In the assessment of patients with RA, many measures are used to assess disease severity including elevated erythrocyte sedimentation rate (ESR), C-reactive protein (CRP), swollen/tender joint count at onset, rheumatoid factor, antibodies to citrullinated proteins, extra-articular features or erosions at presentation (reviewed in [15]). Imaging techniques, in particular power Doppler ultrasound (PDUS), are increasingly being used as imaging biomarkers of RA synovial inflammation. PDUS measures the amplitude of flow signals within blood vessels with high sensitivity [16]. Persistent positive PDUS signal in RA joints predicts development of erosions in both early and established disease [17], [18]. Furthermore, PDUS scores reflect changes in disease activity in patients treated with TNFα antagonists, and patients with reduced PDUS scores following therapy have reduced joint damage progression [19], [20]. However, no data are available that provide a link between PDUS scores and T cell-mediated immunopathology in RA.

This study investigated the presence of Th1, Th17, IL-17+ IFNγ+ and TNFα producing CD4+ T cells in PB, SF and ST from RA patients and related these data to PDUS scores of either single or multiple joints. Our data show that the frequency of IL-17+ IFNγ-CD4+ T cells (Th17), but not IFNγ+IL-17- CD4+ T cells (Th1) or TNFα producing CD4+ T cells, in RA SF positively correlates with systemic inflammation and local PDUS scores. Patients with higher levels of SF IL-17+ CD4+ T cells also show increased levels of the angiogenic factor VEGF-A in SF. In addition, IL-17 and TNFα, but not IFNγ, increase VEGF-A production by RA synovial fibroblasts *in vitro*. In contrast, whilst the percentage of Th17 cells is increased in the blood of RA patients versus healthy controls, this is not correlated with clinical parameters of disease or PDUS.

## Methods

### Objectives

This study aimed to compare frequencies of Th17, Th1, IL-17+ IFNγ+ and TNFα+ CD4+ T cells in PB from RA patients and healthy controls, and to assess if the relative proportions of these cells were increased in either RA SF or ST. Furthermore, we aimed to determine correlations between frequencies of these cells in PB or SF, and synovitis as defined by PDUS score.

### Participants

PB was obtained from patients with established RA according to the 1987 American College of Rheumatology criteria [21] attending for follow-up at Guy's & St Thomas' Rheumatology Department. Healthy controls were recruited from hospital/university students and members of staff. Patients with active arthritis of one or more knees also had SF aspiration following ultrasound of the knee joint. ST was obtained from six patients with RA from the knee or elbow joint during arthroscopy. Age, disease duration, medication, presence or absence of IgM rheumatoid factor or erosions was obtained from review of the medical notes. Disease activity was assessed by DAS28 on the day of sample collection. ESR and CRP were determined on the day of sample collection in the clinical laboratory.

### Cell culture

PB and SF mononuclear cells (PBMC and SFMC, respectively) were isolated by density gradient centrifugation using Lymphoprep (PAA Laboratories, Pasching, Austria) and used immediately. ST samples from the knee or elbow joint were digested using 10 µg/ml collagenase (Sigma-Aldrich, St Louis, USA) in RPMI 1640 (Invitrogen, Paisley, UK) at 37°C for one hour. PBMC, SFMC or ST cells were cultured for 3 hours at a concentration of 1×10^6^/ml in RPMI 1640 medium supplemented with 1% penicillin/streptomycin, 1% glutamine, and 10% heat-inactivated FCS (all PAA). Cultures were stimulated with PMA (50 ng/ml; Sigma-Aldrich) and ionomycin (750 ng/ml; Sigma-Aldrich) with GolgiStop (according to manufacturer's instructions (Becton Dickinson, Franklin Lakes, USA) to encourage accumulation of cytokines intracellularly.

Fibroblasts were cultured from RA ST obtained at the time of arthroplasty until confluent in RPMI 1640 supplemented with 1% penicillin/streptomycin, 1% glutamine, 10% FCS, 0.81X MEM Non-essential amino acids, and 0.81 mM Sodium Orthopyruvate (all Sigma-Aldrich). Once confluent, cells were trypsinized (Trypsin-EDTA, Sigma-Aldrich) and seeded into culture plates. Cells were harvested after five to seven passages for addition of recombinant IL-17, TNFα, or IFNγ (R&D Systems, Minneapolis, USA) at the indicated concentrations.

### Flow Cytometry

Cells were fixed in 2% paraformaldehyde (Merck) prior to permeabilisation with Saponin 0.5% (Sigma-Aldrich) and staining for 30 minutes at 4°C with anti-CD3-PE/Cy7 (Biolegend, San Diego, USA), anti-CD4-PerCP/Cy5.5 (BD), anti-IFNγ-FITC, anti-TNFα-APC (both eBiosciences, San Diego, USA), and anti-IL-17-PE (Biolegend) or the appropriate isotype controls. Cells were acquired on a FACSCanto or FACSCalibur (BD) using bead compensation and analysed using FlowJo (Treestar Inc, Ashland, USA). Live cells were gated using forward and side scatter profiles; T cells were identified by CD3 expression. T helper cell (CD4+ T cell) subsets were defined as follows: Th17 cells were CD3+CD4+IL-17+IFNγ- and Th1 cells were CD3+CD4+IFNγ+IL-17-.

### ELISA

Serum, SF and fibroblast culture supernatants were stored at −80°C prior to analysis. VEGF-A and IL-6 specific ELISA (R&D Systems) was performed according to the manufacturer's instructions. Detection limit of the assay was 31.25 pg/ml for VEGF-A and 20 pg/ml for IL-6.

### PDUS image acquisition and analysis

Ultrasound (US) scans were performed within 2 hours of clinical assessment in a darkened room using a Logiq 9 (GE Healthcare, Buckinghamshire, UK) with a matrix array transducer (5–12 MHz). Synovial blood flow was evaluated by PDUS at the metacarpophalangeal (MCP) joints and both wrists (24 RA patients) and knee joints (20 patients, 22 knees) in longitudinal and transverse views. For the knee joint, longitudinal and transverse images were recorded of the suprapatellar pouch and the lateral and medial patella recesses. The ultrasonographer was blinded to clinical data at the time of the ultrasound examination, and ultrasound images were anonymised prior to scoring. Pulse repetition frequency was adjusted to the lowest permissible value to maximize sensitivity, with the gain set at the point where cortical PDUS signals disappeared. PDUS signals as a percentage of synovial tissue were graded on a semi-quantitative scale from 0 to 3 (where 0  =  no synovial flow; 1  =  up to 25% synovial tissue signal; 2 25–50% signal; 3 >50% signal). Scores for the three areas of a single knee joint were used to calculate a mean knee Doppler score. If fluid was aspirated from both knee joints, then both knee joints were scored independently. The maximum semi-quantitative scores for MCP joints and both wrists (12 joints) were averaged to produce a “hand Doppler” score.

### Ethics

Ethical approval was obtained from the Bromley and St Thomas' NHS Research Ethics Committees, and all patients and controls gave written informed consent.

### Statistical methods

Values are expressed as mean and standard deviations for normally distributed data, or median and interquartile range for non-parametric data. Data were assessed for normality using D'Agostino & Pearson omnibus normality test. Comparisons between patients and healthy controls were made using unpaired t tests or Mann Whitney U tests for parametric and non-parametric data, respectively. Paired PB/SF or ST samples were analysed using either paired t or Wilcoxon signed rank matched pairs tests. Correlation coefficients were obtained using Spearman's method. Data were analysed using Prism version 5 (GraphPad Software Inc, La Jolla, USA). For all tests, p values of less than 0.05 were considered significant.

## Results

### Th17 but not Th1 cells are elevated in peripheral blood of RA patients

We first established whether IL-17, IFNγ, or TNFα producing CD4+ T cells predominate in the PB of established RA patients vs. age and sex matched healthy controls. PB samples were analysed from 38 RA patients and 30 healthy control donors (Table 1). 29 patients (76%) were receiving therapy with either DMARDs or anti-TNF. Of the remaining 9 patients, 4 were DMARD naïve, and 5 had discontinued therapy due to adverse events.

**Table 1:** Characteristics of patients with RA.

	**N = 38**
Age, Mean ± SD	55.3±15.8
Female Sex, n (%)	27 (69)
Disease Duration in years, Median (IQR)	6 (2–10)
Rheumatoid Factor Positive, n (%)	28 (71.8)
Erosive Disease, n (%)	22 (56)
DAS28, Mean ± SD	4.6±1.5
ESR, mm/hour, Median (IQR)	20 (11.5–33)
CRP, g/dl, Mean ± SD	14±14.6
DMARDs (excluding anti-TNF), n (%)	18 (47)
Anti-TNFα therapy, n (%)	11 (29)
Systemic steroids, n (%)	8 (21)

Normally distributed data are represented as mean ± standard deviation, otherwise as median with inter-quartile range. Mean age of 30 healthy controls (12 males/18 females) was 51±20 years (age and sex not statistically significant compared to RA group).

**Figure 1:**
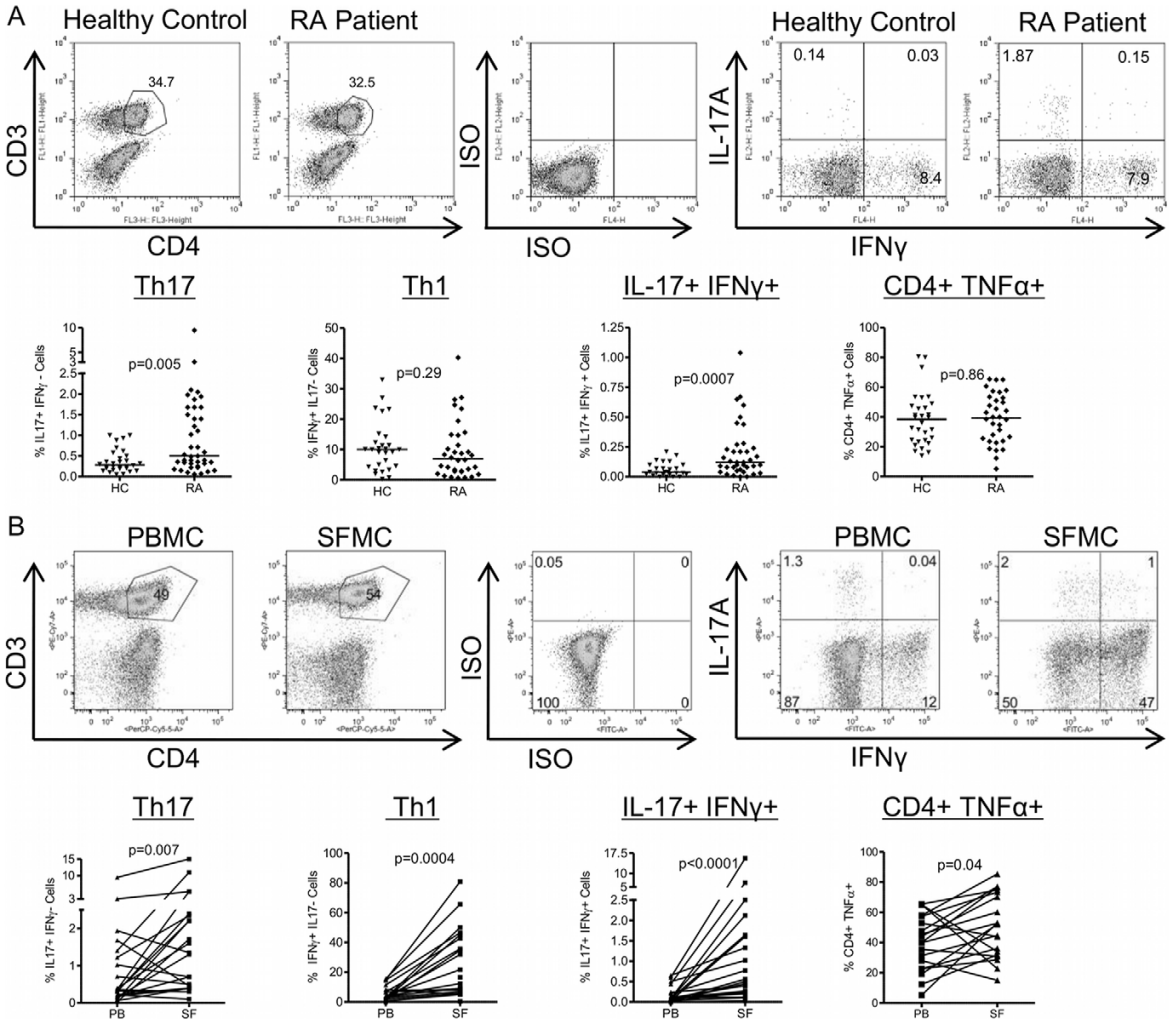
Presence of Th17, Th1, IL-17+IFNγ+ and TNFα-expressing CD4+ T cells in RA PB and SF. Mononuclear cells were isolated from PB from 38 RA patients and 30 healthy controls (A), or paired RA PB and SF samples (n = 22) (B). Cells were stimulated ex vivo with PMA and ionomycin for 3 hours prior to intracellular staining for CD3, CD4, IL-17, IFNγ and TNFα. Upper panels in (A, B) show representative gating strategy: Live cells were gated using forward and side scatter profiles, then CD4+ T cells gated based on their expression of CD3 and CD4, and the intracellular cytokine expression determined. Lower panels in (A, B) show scatter plots of the percentages of IL-17+IFNγ- (Th17 cells), IFNγ+IL-17- (Th1 cells), IL-17+IFNγ+ CD4+ T cells and total TNFα+ CD4+ T cells as a percentage of CD4+ T cells. Each symbol/line represents an individual donor. (A) The percentage of cytokine expressing cells in PB from healthy control vs. RA patients. Horizontal bar represents median value. Comparisons between groups were made using Mann-Whitney U tests. (B) The percentage of cytokine expressing cells in paired RA PB vs. SF samples. Comparisons were made using Wilcoxon matched pairs tests.

*Ex vivo* (PMA/ionomycin/GolgiStop stimulated for 3 hours) Th17 cell frequencies were significantly elevated in RA patients vs. healthy donors (HC median 0.32, inter-quartile range (IQR) 0.21–0.54%, RA median 0.5, IQR 0.32–1.68%, p = 0.005), with a suggestion of a bimodal distribution (Fig. 1A) in patients. Cells expressing both IL-17 and IFNγ were also observed at significantly higher frequencies in RA patients. There were no significant differences in the frequency of Th1 or TNFα-expressing CD4+ T cells between RA patients and healthy controls, however these cells were present at higher frequencies than Th17 cells.

### Th17, Th1, IL-17+IFNγ+ and TNFα expressing CD4+ T cells are increased at the site of inflammation in RA

To determine whether Th17 cells are also increased at the site of inflammation, we compared frequencies of Th17, Th1, IL-17+IFNγ+ and TNFα+ CD4+ T cells in SF (22 knees) with paired PB from 20 patients with active RA. SF was obtained following PDUS of an inflamed knee joint. Frequencies of Th17 cells were significantly increased in SF relative to paired PB (Fig. 1B). We also found highly significant increases in Th1 and IL-17+IFNγ+ CD4+ T cells, and an increase in TNFα-producing CD4+ T cells between the two compartments. Some of this increase may be explained by an increase in the CD4+ memory T cell fraction in SF vs. PB; however the mean ± SD fold increase in Th17 cells from PB to SF was 6.6 ± 12.7 fold (range 0.2–57 fold) whereas the CD45RO proportion changes two-fold, from 50% in PB to 90–95% in SF [22] (NG & LT, unpublished data).

**Figure 2:**
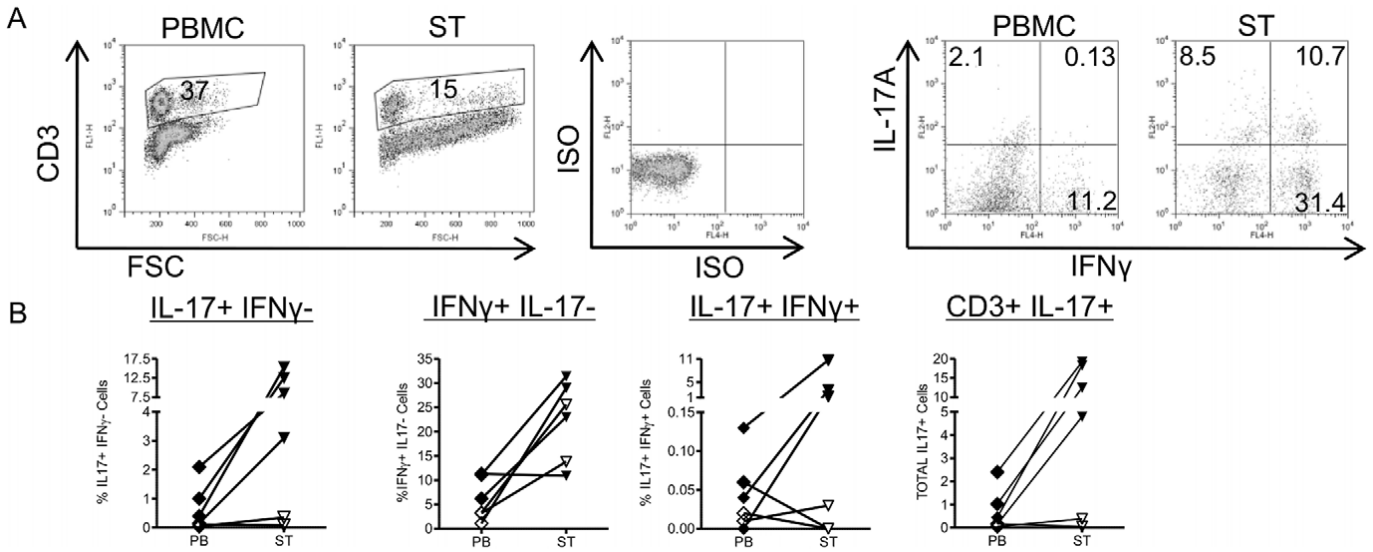
IFNγ and IL-17-producing T cells are increased in synovial tissue from patients with active RA. Mononuclear cells were isolated from PB. Synovial tissue (ST) samples were obtained at arthroscopic synovial biopsy and digested with collagenase prior to stimulation and staining as described in Fig. 1. Stains for TNFα were not performed. A) T cells were gated based on CD3 expression, and intracellular cytokine expression determined. B) Scatter plots of PB vs. ST, showing percentages of IL-17+IFNγ-, IFNγ+IL-17-, IL-17+IFNγ+ and total IL-17+ T cells as a percentage of CD3+ T cells (n = 6). Closed symbols: patients with active disease (DAS28>3.2); open symbols: patients in remission (DAS28<2.6).

**Figure 3:**
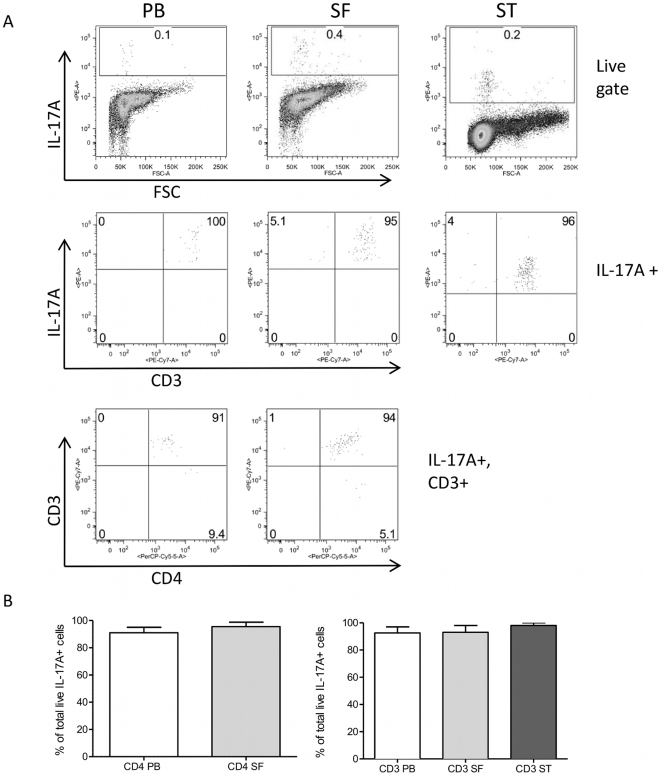
IL-17+ cells from RA PB, SF and ST are predominantly CD3+ T cells. A) PB (n = 18), SF (n = 10) and ST (n = 6)-derived cells were prepared as described in the Methods, and live cells were gated using forward and side scatter profiles. IL-17+ cells were identified based on isotype control staining, and the percentage of either CD3+ or CD3+CD4+ T cells within the IL-17+ gate determined for each sample. B) Bar graphs showing mean and SD of the percentage CD4+ T cells within the IL-17+ population for PB and SF, and of the percentage CD3+ T cells within the IL-17+ population for PB, SF and ST.

IL-17 as well as IFNγ producing T cells were also detected in ST from a small group of patients undergoing arthroscopic biopsy (Fig. 2). In agreement with data from other groups [12], [23], not all patients had equally high levels of IL-17+ T cells. Interestingly, of six patients, the four patients with active disease (DAS28>3.2) had a much higher percentage of IL-17+IFNγ- T cells in ST (3–15%) compared to the two patients that were in remission (DAS28<2.6; <0.4%). A similar trend was not seen for IFNγ producing cells. These findings suggest a relationship between local IL-17 expression and active synovitis. Of note, in our studies, the vast majority of cells producing IL-17 in PB, SF or ST were T cells (% CD3+ mean ± SD in PB 95±4.5; SF 95±4.5; ST 95.6±7.8%, Fig. 3).

### Presence of Th17 cells in synovial fluid is positively correlated with C-reactive protein and local PDUS score

We then assessed if the presence of CD4+ T cells expressing IL-17, IFNγ or TNFα in SF was correlated with systemic markers of inflammation (Table 2). The frequency of Th17 cells in SF was positively correlated with CRP (r = 0.51, p = 0.04, n = 20) whilst no correlations were observed between systemic markers of inflammation and Th1 cell frequency. Surprisingly, the frequency of TNFα-producing CD4+ T cells in SF was negatively correlated with both DAS28 and ESR. In contrast, there were no correlations between systemic markers of inflammation and inflammatory cytokine-expressing CD4+ T cells in PB.

**Table 2:** Correlations between frequencies of Th17, Th1, IL-17+IFNγ+ and TNFα expressing CD4+ T cells in synovial fluid and peripheral blood and DAS28, ESR and CRP.

	DAS28		ESR		CRP	
**SF (n = 22)**	**r**	**p**	**r**	**p**	**r**	**p**
Th17	0.16	0.49	0.27	0.49	**0.51**	**0.04**
Th1	−0.21	0.34	−0.36	0.34	−0.16	0.84
IL-17+IFNγ+	0.06	0.79	0.08	0.76	0.31	0.28
CD4+ TNFα (n = 18)	**−0.70**	**0.002**	**−0.65**	**0.008**	−0.48	0.10
**PB (n = 38)**						
Th17	0.10	0.53	−0.14	0.40	0.07	0.70
Th1	0.08	0.63	−0.13	0.46	0.06	0.73
IL-17+IFNγ+	0.10	0.56	−0.16	0.34	−0.12	0.51
CD4+ TNFα (n = 32)	−0.13	0.5	−0.1	0.6	−0.21	0.29

Correlations were performed using Spearman's rank correlation. Statistically significant correlations are indicated in bold.

**Figure 4:**
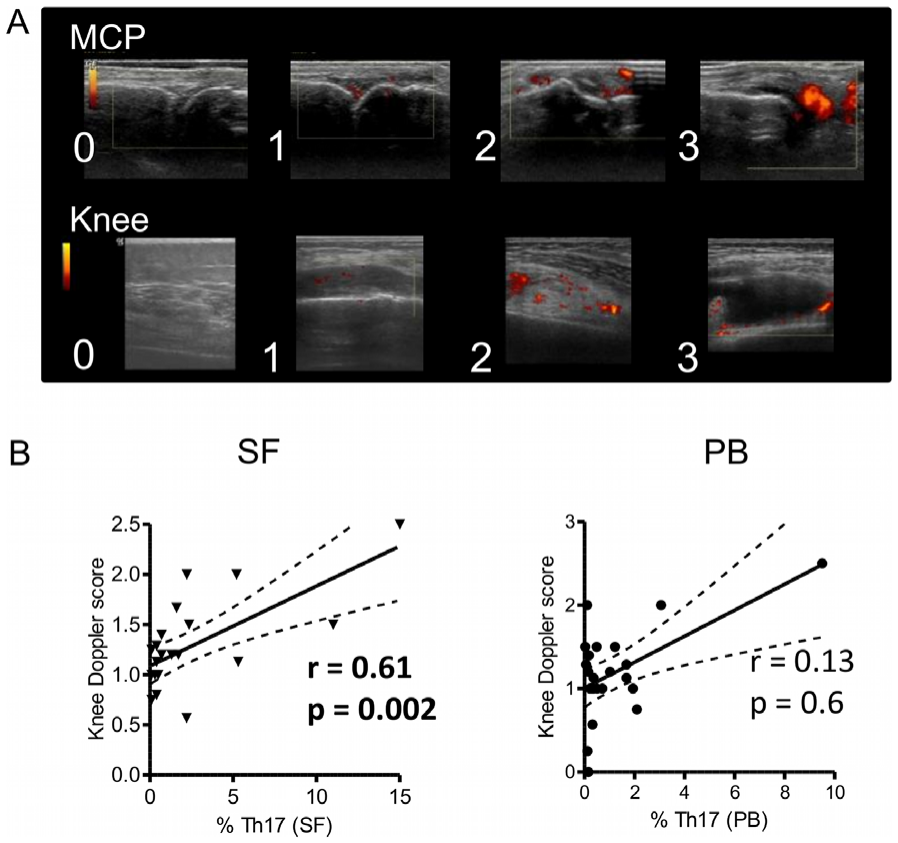
Single knee PDUS scores are highly correlated with the presence of Th17 cells in SF. A) Representative images for semi-quantitative ultrasound scoring (MCP, upper panel; knee, lower panel). B) Correlations between knee PDUS scores and the percentage of Th17 cells in SF vs. PB (n = 22). The regression lines with 95% confidence intervals are shown.

PDUS is increasingly used as an imaging biomarker of RA synovial inflammation, and has been associated with increased joint damage progression in RA [18], [19]. Histopathological studies suggest association of positive PDUS signal with neoangiogenesis [24]. However, cytokine expression within the joint has not been associated with increased PDUS signal thus far. Patients with RA had ultrasound of either the MCP joints and wrists, or a swollen knee joint (prior to aspiration of SF). Images were scored semi-quantitatively on a scale 0–3 as described in the methods (representative images in Fig. 4). All knee joints had mean Doppler >0, i.e. all joints scanned had a positive Doppler signal on at least one image. These patients were all symptomatic with active disease clinically, requiring joint aspiration. The patients undergoing hand joint PDUS included a wider cross-section of disease activity: 4 patients had no active synovitis by PDUS, 7 patients had at least 6 out of 12 joints with positive Doppler. The remaining patients had up to 5 out of 12 positive joints. Overall, the mean number of hand joints with positive Doppler (± SD) was 3.7±3.1; with an average mean semi-quantitative PDUS score of 0.47±0.46. The validity of PDUS as a synovitis biomarker was confirmed by our findings that PDUS scores of multiple hand joints showed strong positive correlations with disease activity (DAS28, r = 0.64, p = 0.0005, n = 26) and inflammatory markers (ESR, r = 0.54, p = 0.005, n = 26; CRP r = 0.48, p = 0.02, n = 24). PDUS of a single knee joint was also positively correlated with DAS28 (r = 0.51, p = 0.02), ESR (r = 0.48, p = 0.02, both n = 26), and CRP (r = 0.49, p = 0.04, n = 24). When the relationship between PDUS scores and inflammatory cytokine expressing CD4+ T cell frequencies was investigated, a positive correlation was observed between SF Th17 cell frequencies and the local PDUS of the same knee joint (r = 0.61, p = 0.002; n = 22; Fig. 4B and Table 3). We also found a positive correlation between knee PDUS and IL-17+IFNγ+ CD4+ T cell frequency in SF (r = 0.52, p = 0.04; n = 22). These relationships were not observed for either Th1 or TNFα expressing CD4+ T cells in SF, or for Th17, Th1, IL-17+IFNγ+ or TNFα-producing CD4+ T cells from PB (Fig. 4B, Table 3). PDUS data were also available for five patients undergoing ST biopsies. Patients in remission (n = 2, with low levels of IL-17+ cells) had mean PDUS scores of <1.0, whilst patients with active disease (n = 3) had PDUS>1.5. Together, these data indicate that PDUS-defined active synovitis is associated with the presence of local IL-17-producing CD4+ T cells. This finding provides novel insight into the immunopathological mechanisms of PDUS-related joint damage progression.

**Table 3:** Correlations between local knee PDUS score and frequencies of Th17, Th1, IL-17+IFNγ+ and TNFα expressing CD4+ T cells in SF vs. PB.

	Knee PDUS scoreSpearman correlation (n = 22)	
**SF**	r	p
Th17	**0.61**	**0.002**
Th1	−0.04	0.61
IL-17+IFNγ+	**0.52**	**0.04**
CD4+ TNFα (n = 18)	−0.14	0.59
**PB**		
Th17	0.12	0.57
Th1	−0.03	0.91
IL-17+IFNγ+	−0.08	0.69
CD4+ TNFα (n = 18)	0.20	0.43

Correlations were performed using Spearman's rank correlations. Statistically significant correlations are indicated in bold.

### Th17 cells in synovial fluid are associated with increased VEGF-A production

**Figure 5:**
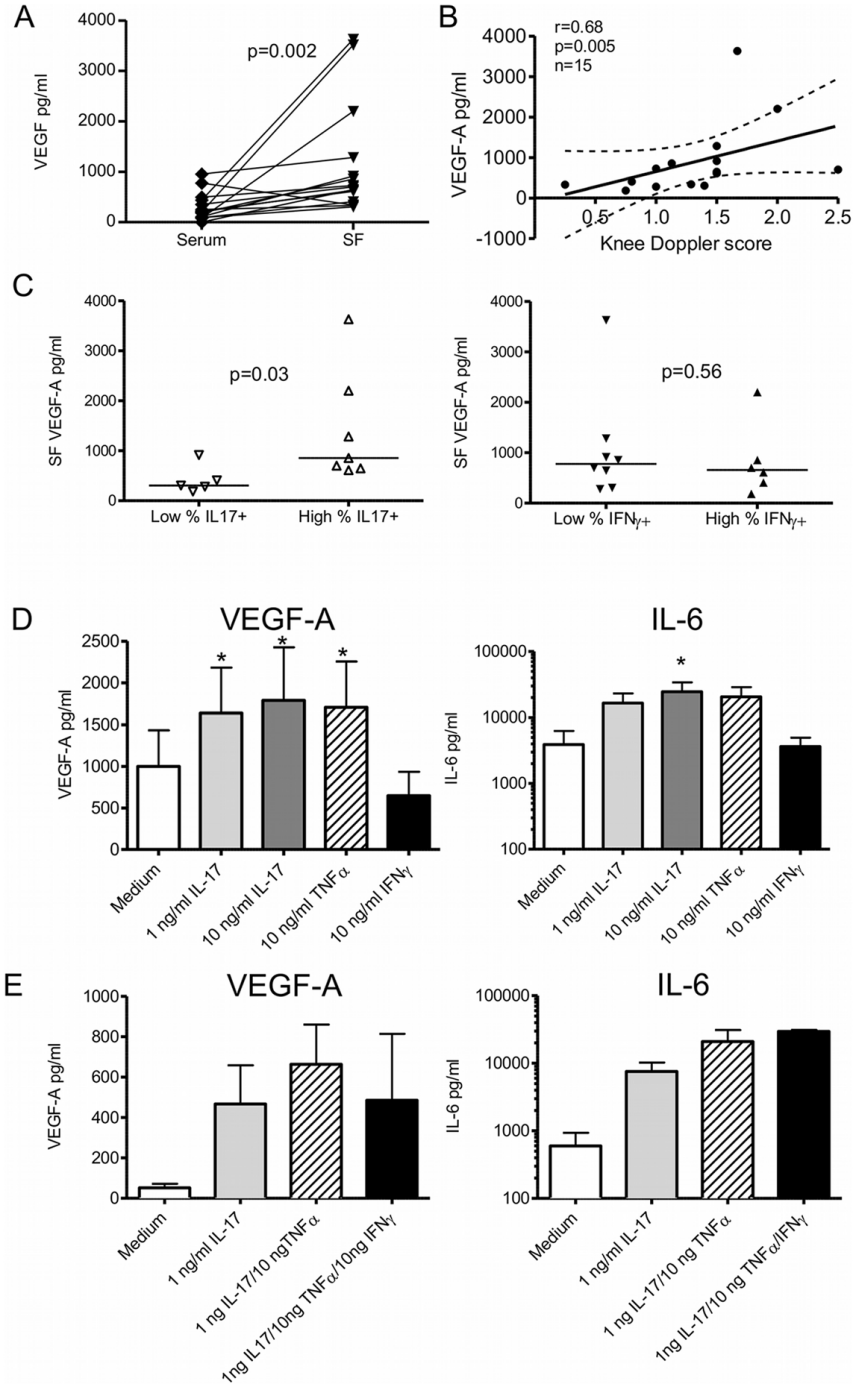
The presence of Th17 cells in SF is linked to increased VEGF in SF. A) VEGF-A levels in cell-free RA SF and matched paired serum were determined by ELISA (n = 15). B) Scatter plot of VEGF-A levels in SF vs. knee PDUS score, and the corresponding Spearman correlation coefficient and p-values. The regression line and 95% confidence intervals are shown. C) SF VEGF-A levels in RA patients stratified based on their frequency of total IL-17+ CD4+ T cells or total IFNγ+ CD4+ T cells (below or above the median level). Groups were compared using Mann Whitney U tests. (n = 12) D) RA synovial fibroblasts (n = 6) were cultured for 48 hours in the presence of 1 ng/ml rIL-17, 10 ng/ml rIL-17, 10 ng/ml rTNFα or 10 ng/ml rIFNγ. VEGF-A and IL-6 were measured in culture supernatants by ELISA. Comparison between groups was made using one-way ANOVA; * p<0.05. E) RA synovial fibroblasts (n = 3) were cultured for 48 hours alone or with a combination of 1 ng/ml rIL-17, 10 ng/ml rTNFα, and 10 ng/ml rIFNγ prior to measurement of VEGF-A and IL-6 in supernatants.

Angiogenesis is a key process in joint swelling and inflammation in RA (reviewed in [25]). In our patients, the angiogenic factor VEGF-A was significantly elevated in SF relative to paired serum (Fig. 5A, n = 15). Since PDUS is a sensitive method of assessing blood flow within tissues, we found as expected, that knee PDUS scores correlated strongly with the presence of VEGF-A in SF (Fig. 5B). In keeping with our observed associations between the presence of Th17 and IL-17+IFNγ+ CD4+ T cells in SF and local PDUS signal, we found that patients with high frequencies of total IL-17+ CD4+ T cells in SF (above the median level) had significantly higher levels of SF VEGF-A. In contrast, there was no significant difference between SF VEGF-A in patients with high vs. low IFNγ+ CD4+ T cells in SF (Fig. 5C, n = 12).

To demonstrate a causal relationship between the presence of IL-17 producing cells within the joint and VEGF-A production, we cultured synovial fibroblasts (a known source of VEGF-A [26]) from 6 patients with RA with rIL-17 and measured secreted VEGF-A (Fig. 5D). As a control for the presence of Th1 or TNFα producing T cells, we set up parallel experiments with rIFNγ and rTNFα. Some RA synovial fibroblasts spontaneously produced VEGF-A, however VEGF-A production was significantly increased by rIL-17 even at low concentrations (1 ng/ml). rIL-17 at higher concentrations also significantly induced IL-6 secretion by RA synovial fibroblasts, a cytokine involved in both inflammation and angiogenesis (reviewed in [27]). Similar increases in VEGF-A production were seen upon addition of rTNFα. No evidence was observed for synergy between rTNFα and rIL-17 (Fig. 5E). Addition of rIFNγ did not induce VEGF-A or IL-6 secretion, and in fact reduced VEGF-A levels and IL-6 in 4 out of 6 fibroblast lines. Given that IL-17, TNFα as well as IFNγ producing T cells are simultaneously present in the RA joint (Fig. 1, 2), we assessed the effect of the combined cytokines on VEGF-A and IL-6 production. In all three lines tested, a clear increase in VEGF-A as well as IL-6 was observed (Fig. 5E), indicating that the potentially antagonistic effect of rIFNγ was overcome by rIL-17 and rTNFα. Together, these data are highly suggestive of a role of local IL-17 in stimulating VEGF-A production and angiogenesis.

## Discussion

In this paper, we demonstrate that Th17, Th1 and IL-17+IFNγ+ CD4+ T cells as well as TNFα-expressing CD4+ T cells are enriched within RA SF relative to paired PB. The frequency of Th17 cells in SF correlates significantly with CRP, a marker for systemic inflammation, suggesting that local Th17 cells contribute to active disease. In contrast, although Th1 cells greatly outnumbered IL-17+ T cells in SF, they did not correlate with markers of inflammation. These data support the DAMAGE study, which showed that synovial tissue IL-17 mRNA levels strongly correlate with CRP, and in synergy with TNFα, predict rapid joint damage progression in RA [23]. Importantly, we also detected IL-17+ T cells in ST, but only at significant levels in those patients that had active RA, suggesting that IL-17+ T cell numbers may vary with disease activity (Fig. 2). Other studies also report that IL-17+ T cells or IL-17 mRNA are not universally present in RA ST, and are found in 28–60% of RA ST samples [12], [23], [28]. A recent paper reported that mast cells rather than T cells are the major source of IL-17 in RA ST [29]. In our experiments the vast majority of cells in PB, SF and ST producing IL-17 were T cells, although our protocol used T cell stimulation with PMA and ionomycin. In addition, Stamp et al did not detect IL-17 production by other cell types [12]. One explanation for these different observations may be that in the study by Hueber *et al*. ST was obtained at the time of joint replacement, where tissue responses to joint damage may predominate over active inflammation. Indeed, high levels of mast cells are observed in avascular, fibrotic regions of RA synovial tissue, without correlations with lymphocytic infiltration [30].

Both synovial tissue IL-17 mRNA expression and persistent PDUS signal have been independently associated with increased joint damage progression in RA [18], [19], [23]. Histopathological studies suggest association of positive PDUS signal with neoangiogenesis [24]. However cytokine expression within the joint has not been associated with increased PDUS signal thus far. Our demonstration that the local presence of IL-17 producing CD4+ T cells (either Th17 or IL-17+IFNγ+ T cells) in the RA joint is associated with active synovitis measured by PDUS, provides novel mechanistic insight into PDUS-related immunopathology. Through their production of IL-17 and TNFα, Th17 cells can promote osteoclastogenesis [31] and cartilage damage [32], [33] in the RA joint, thus accelerating joint damage progression. In addition, we demonstrate that IL-17 and TNFα promote VEGF-A production by RA synovial fibroblasts. A role for IL-17 in angiogenesis is supported by the recent findings that local overexpression of IL-17 in C57BL/6 mice induces arthritis with increased vascularity, and that IL-17 induces migration of endothelial cells *in vitro*, along with increased angiogenesis in Matrigel plugs [34]. In contrast, we found that IFNγ did not induce VEGF-A secretion by RA synovial fibroblasts, which is in agreement with the lack of correlation between local Th1 cell frequency and PDUS signal. The lack of correlation with IFNγ is in agreement with the findings that IFNγ mRNA levels in ST were strongly negatively correlated with joint damage progression [23], and that Th1 cells inhibit development of osteoclasts [31].

Highly vascularized synovium (indicated by positive PDUS signal) is likely to permit increased influx of inflammatory cells into the joint, and one explanation for our findings could therefore be that Th17 cells are elevated simply as a bystander effect. However, the lack of correlation between the frequency of Th1 cells or TNFα+ CD4+ T cells with PDUS scores argues against this explanation. In contrast to non-specific influx, Th17 cells may preferentially migrate into the joint via CCR6 interaction with CCL20, which is strongly expressed in the synovium [35]. In addition, we have previously proposed a mechanism for generation of Th17 cells in the joint itself, as cell contact with *in vitro* (LPS) activated [36] or *in vivo* activated monocytes from inflamed joints from RA patients [11] leads to induction of IL-17 production in memory CD4+ T cells, suggesting that newly recruited CD4+ T cells can be skewed toward a Th17 phenotype at the site of inflammation.

Despite the fact that we found significant increases in Th17 and IL-17+IFNγ+ CD4+ T cells in the blood of RA patients vs. healthy donors, these subsets did not correlate with markers of disease such as ESR, CRP or DAS28. This suggests that the presence of IL-17 producing CD4+ T cells in the blood from patients with established RA is of limited use as a biomarker to indicate disease activity. A significant increase in the percentage of IL-17+ CD4+ T cells in PB of RA patients vs. healthy donors was recently also described by Shen *et al*. [37]. In contrast, two separate studies reported no differences in Th17 cell frequency in PB of RA patients vs. healthy controls [28], [38]. All three studies, however, concur with ours that no differences are detected in the percentage of Th1 cells in RA vs. healthy donors. It is currently unclear why the observations regarding Th17 cell frequencies in RA are more varied, but this may be due in part to different demographics and treatment regimes in the patient populations. Indeed, it is worth noting that in all these studies the focus has been on RA patients with established disease, treated with a wide variety of therapies, which in combination with changes in disease activity could influence PB Th17 levels and their correlations with markers of disease activity. This idea is supported by a recent study involving patients with early, treatment-naive RA and psoriatic arthritis where systemic disease activity in the early disease stages strongly correlated with the frequency of Th17 cells within PB [39].

Our finding that TNFα expressing CD4+ T cells in SF were negatively correlated with DAS28 and ESR is perhaps surprising given the well-documented responses to TNFα antagonists in RA [40], [41], [42]. However, we analysed expression of TNFα by CD4+ T cells only, whilst TNFα is expressed by many other cells in rheumatoid synovium, in particular cells of the monocyte-macrophage lineage [43]. There are few studies documenting the relationship between disease activity and expression of TNFα in the joint, and these studies have used TNFα expression in synovial tissue. Tak *et al* showed a strong correlation between semi-quantitative histology scores for TNFα and knee pain but no correlations were reported for either systemic inflammatory markers or global disease activity [44]. Morand *et al* found no correlations between synovial lining layer TNFα and measures of disease activity, but noted reduced TNFα following successful therapy with DMARDs [45]. Clearly further research relating markers of disease activity to TNFãexpression in T cells vs. monocytes/macrophages and fibroblasts from synovial fluid vs. tissue is still required.

### Limitations

We defined Th17 and Th1 cells on the basis of cytokine expression by CD4+ T cells rather than presence or absence of specific transcription factors such as RORc2 or T-bet. Although this approach is widely used in the field (e.g. [28], [39], [46], [47]), one should remain aware that our study describes specific cytokine profiles of CD4+ T cells rather than cell lineage.

As we aimed to obtain ST from arthroscopic biopsies rather than from patients undergoing joint replacement to prevent confounding by fibrotic tissue findings, only a relatively low number of synovial tissue samples were available to investigate if remission is associated with low levels of IL-17+ T cells. One possibility could have been to extend the dataset with SF data, however all of our patients requiring SF aspiration had active disease. Interestingly though, patients with low disease activity (DAS28<3.2) had significantly lower Th17 cells in SF than patients with active disease (DAS28>3.2) (median 1.5 vs. 0.35%, p = 0.04, n = 22, data not shown), lending further weight to the associations between systemic inflammation, local synovitis and Th17 cell frequency.

Our data raise a number of other questions regarding the presence of Th17 cells in RA. We have observed a relationship between local, but not peripheral blood Th17 cells and active synovitis. This relationship may be more pronounced at earlier stages of disease, with IL-17 having a greater contribution towards joint damage progression [23]. It is now clear that some experimental arthritis models are dependent on Th1/IFNγ [48], whilst others are Th17/IL-17 dependent [49] or switch from TNFα dependency to a Th17/IL-17 profile in later stages of disease [50]. This raises the possibility that in RA T helper cell subsets may play different roles at different stages of disease or depending on genetic background of patients, which may affect disease activity or outcome.

Furthermore, in a cross-sectional study such as ours, we are unable to conclude if the relationship with active disease activity is dynamic; i.e. are Th17 cells present at times of active disease and disappear when disease is suppressed? In addition, it is intriguing to speculate if the presence of these cells specifically indicates those patients with a predominantly IL-17/Th17-mediated disease from the outset and who may thus benefit from specific therapy with IL-17 antagonists. Interestingly, a number of the patients in this study with high levels of Th17 cells in SF were non-responders to anti-TNF therapy, and this could either indicate that these patients have IL-17-related pathology, or that the presence of Th17 cells in the joint is a marker of poor response to TNFα blockade. Longitudinal studies including ST biopsies before and after IL-17 vs. TNFα antagonists will be required to answer these important questions.

In summary, we have used PDUS as a biomarker of active synovitis to explore relationships between imaging and T cell-mediated immunopathology. This technique could be replicated to investigate contributions of other cell types including monocytes/macrophages to immunopathology in RA. PDUS is increasingly used to identify patients at risk of joint damage progression in the clinic. Our data provide an immunologic explanation for this clinical observation by demonstrating that the presence of the pro-inflammatory and osteoclastogenic Th17 cell subset in the inflamed joint strongly correlates with both disease activity and local PDUS score in RA. Our findings thus highlight PDUS as a possible surrogate marker of Th17 cells, and may identify patients who would benefit from early, aggressive therapy, including IL-17 antagonists.
